# The Effect of Preoperative Magnetic Resonance Imaging on Long-term Oncological Outcomes Following Radical Prostatectomy—A 12-year Follow-up of a Randomized Controlled Trial

**DOI:** 10.1016/j.euros.2025.07.005

**Published:** 2025-08-07

**Authors:** Daniyal Noor, Eduard Baco, Peter M. Lauritzen, Viktor Berge, Kristina F. Galtung, Maciej Jacewicz, Lars Magne Eri, Erik Rud

**Affiliations:** a Division of Radiology and Nuclear Medicine, Oslo University Hospital, Oslo, Norway; b Institute of Clinical Medicine, University of Oslo, Oslo, Norway; c Department of Urology, Oslo University Hospital, Oslo, Norway; d Department of Life Sciences and Health, Faculty of Health Science, Oslo Metropolitan University, Oslo, Norway

**Keywords:** Magnetic resonance imaging, Prostatectomy, Prostatic neoplasms, Randomized controlled trials as topic, Recurrence, Survival rate

## Abstract

**Background and objective:**

The effect of preoperative magnetic resonance imaging (MRI) on long-term oncological outcomes following robot-assisted laparoscopic prostatectomy (RALP) is uncertain. The objective was to investigate the effect of preoperative MRI on long-term oncological outcomes after RALP.

**Methods:**

A single-institution follow-up study of a randomized controlled trial was conducted, which included 438 patients with biopsy-confirmed prostate cancer scheduled for RALP between December 2009 and June 2012 (NCT06429878). No patients underwent MRI prior to biopsy. Patients were randomized into two groups: one without MRI (*n* = 216) and one with preoperative MRI (*n* = 222). The primary endpoints were disease-free (DFS) and overall (OS) survival. The effect of MRI on these endpoints was estimated using hazard ratios (HRs) with 95% confidence intervals (CIs).

**Key findings and limitations:**

The median age was 63 yr (interquartile range [IQR] 59–67), and the median prostate-specific antigen level was 7.9 ng/ml (IQR 5.9–11.4). Based on D’Amico risk classification, 112 patients were at a low risk, 220 at an intermediate risk, and 106 at a high risk. The median follow-up was 111 mo (95% CI: 104–118). The 12-yr DFS rate was 60% (95% CI: 54–65) and OS rate was 87% (95% CI: 84–90). Preoperative MRI did not improve DFS (HR 0.97, 95% CI: 0.71–1.34) or OS (HR 0.86, 95% CI: 0.52–1.40), with similar findings across clinical subgroups. Limitations include the following: oncological outcomes were secondary endpoints and MRI pathway has undergone changes since the trial.

**Conclusions and clinical implications:**

During a 12-yr follow-up after RALP, we did not observe a statistically significant difference in DFS or OS between patients who underwent preoperative MRI and those who did not. These findings highlight the need to assess the long-term impact of prebiopsy MRI.

**Patient summary:**

We studied whether having a magnetic resonance imaging (MRI) scan before prostate cancer surgery improves long-term outcomes. A total of 438 men with prostate cancer were included in this study and they were divided into two groups: one without MRI and one with MRI before surgery. The patients were followed for up to 12 yr, and we found no clear reduction in the risk of cancer recurrence or improved survival in patients who had undergone MRI. Today, MRI is performed routinely before biopsy, and more research is needed to evaluate whether this change has improved long-term outcomes.

## Take Home Message

During a 12-yr follow-up after robot-assisted laparoscopic prostatectomy, no statistically significant difference was observed in disease-free or overall survival between patients who underwent preoperative magnetic resonance imaging and those who did not.

## 1 Introduction

The effect of preoperative magnetic resonance imaging (MRI) on surgical and long-term oncological outcomes in prostate cancer remains unclear. The primary perceived advantage of preoperative MRI is its ability to assess local tumor extent, particularly in detecting extraprostatic extension (EPE). When EPE is suspected, surgeons may opt for wider excision to reduce the risk of positive surgical margins (PSMs), persistent disease, and biochemical recurrence (BCR). Although several studies have evaluated the diagnostic performance of MRI, including cancer detection and staging, evidence regarding its effect on PSM rates, BCR, and overall survival (OS) remains limited and conflicting [1,2].

Between 2009 and 2012, we conducted the only randomized trial to date evaluating whether preoperative MRI could reduce the rate of PSMs in patients scheduled for robot-assisted laparoscopic prostatectomy (RALP) [3]. In this initial study, we observed that patients in the MRI group underwent fewer nerve-sparing surgeries, but without a statistically significant reduction in the PSM rate.

Since the completion of our trial, the MRI pathway in prostate cancer management has changed substantially. MRI is now performed routinely before biopsy, and this approach improves the detection of clinically significant cancers while reducing overdiagnosis [4]. Despite this shift, MRI remains integral for surgical planning and its influence on long-term outcomes remains uncertain.

The present study reports 12-yr follow-up data from our randomized trial, and aims to compare disease-free survival (DFS) and OS in patients randomized to no MRI versus MRI prior to RALP.

## 2 Patients and methods

### 2.1 Study design and participants

This single-institution follow-up study of a randomized controlled trial included 438 patients with biopsy-confirmed prostate cancer scheduled for RALP between December 2009 and June 2012 (ClinicalTrials.gov identifier: NCT01347320). None of the patients underwent MRI before biopsy. Patients were randomized to either no MRI or preoperative MRI. The primary endpoint was PSMs, and the effect of MRI was estimated as the difference in proportions of PSMs between the groups. The study was approved by the regional committee for medical and health research ethics (no.: 248365) and registered at Clinical Trials.gov (NCT06429878).

Patients were allocated (1:1) to either the non-MRI or the MRI group using a permuted block randomization procedure with undisclosed and variable block sizes to minimize predictability. Allocation was concealed using opaque, sealed envelopes, prepared in advance by the Centre of Biostatistics and Epidemiology. The envelopes were accessible only to the principal investigator, who performed the randomization. Each patient, after providing consent, was assigned a unique, sequentially numbered randomization code that matched the envelope. The entire process was documented and overseen by a statistician.

All patients underwent RALP, with or without pelvic lymph node dissection (PLND).

Patients randomized to preoperative MRI underwent a biparametric MRI protocol consisting of high-resolution three-dimensional (3D) T2-weighted and axial diffusion-weighted images. The MRI report included tumor location and size, and the presence of T3 disease, defined as EPE and/or seminal vesicle invasion. The index tumor was defined as the largest tumor or the tumor associated with T3 disease. Full details on trial design, eligibility criteria, and methodology can be found in a prior publication [3].

### 2.2 Endpoints

The primary endpoints of this follow-up study were DFS and OS.

### 2.3 Statistical analysis

All analyses adhere to the intention-to-treat principle. After surgery, patients were considered prostate-specific antigen (PSA) free if at least one PSA value was <0.1 ng/ml within the first 3 mo. BCR was defined as PSA ≥0.2 ng/ml on at least two occasions, after first being PSA free. Persistent disease was defined as PSA not reaching <0.1 ng/ml within the 3 mo, followed by PSA ≥0.2 ng/ml on at least two occasions.

The median follow-up time was estimated using the reverse Kaplan-Meier method. DFS was defined as the number of months from surgery to a diagnosis of persistent disease or BCR. Patients who were lost to follow-up before experiencing BCR were censored at the date of their last known recurrence-free PSA control.

OS was measured in months from surgery to the date of death. Data were censored on July 7, 2023, when we confirmed the vital status of all patients using the national electronic death registry as the reference.

The effect of MRI on the primary endpoints was estimated using hazard ratios (HRs) with 95% confidence intervals (CIs). Survival curves were expressed according to the Kaplan-Meier method, and any difference between the non-MRI and MRI groups was assessed by the log-rank test. Prespecified subgroup analyses were conducted to assess the effect of MRI on DFS and OS across clinically relevant subgroups. These subgroups included pathological tumor stage (pT2 and pT3), D’Amico risk classification (low, intermediate, and high risk), Gleason grade group in the prostatectomy specimen (groups 1–5), and clinical tumor stage (cT1–cT3). For each subgroup, univariate Cox proportional hazard models were used to estimate HRs and 95% CIs. Differences in HRs across clinical subgroups were assessed by using an interaction term between the MRI groups and the clinical subgroups.

We conducted statistical analyses using IBM SPPS Statistics version 25.0 (IBM Corp., Armonk, NY, USA) and MedCalc statistical software version 14.8.1 (MedCalc Software, Ostend, Belgium).

## 3 Results

At the time of surgery, the median age was 63 yr (interquartile range [IQR] 59–67), and the median PSA was 7.9 ng/ml (IQR 5.9–11.4). Of the 438 patients included, 216 were randomized to the non-MRI group and 222 to the MRI group. Table 1 summarizes the preoperative characteristics of both groups.

**Table 1:** Preoperative characteristics of 438 patients randomized to the non-MRI or MRI group before radical prostatectomy

	Non-MRI (*n* = 216)		MRI (*n* = 222)	
	Median	IQR	Median	IQR
Age at surgery (yr)	64	59–67	63	58–67
Preoperative PSA (ng/ml)	8.2	6–12	7.8	6–11
Prostate volume (ml)	37	31–48	37	28–47
Body mass index (kg/m^2^) a	26	24–28	26	24–28
	*n*	%	*n*	%
D’Amico risk group				
Low	55	25	57	26
Intermediate	103	48	114	51
High	58	27	51	23
Gleason grade group in biopsy				
1	79	37	70	32
2	79	37	89	40
3	20	9	27	12
4	37	17	28	13
5	1	0.5	8	3

IQR = interquartile range; MRI = magnetic resonance imaging; PSA = prostate-specific antigen.

a Missing values in 39 patients.

We have previously reported on MRI findings and their impact upon surgical planning. In summary, MRI identified the index tumor in 92% of cases, with sensitivity of 73% and specificity of 65% for detecting extraprostatic disease [5,6]. The surgeons reported that MRI influenced the surgical technique on one or both sides in 27% of cases, with all adjustments resulting in more radical excisions [3]. There was a radiological T3 stage in 83% of cases where surgeons reported adjustments in the surgical approach based on MRI. There was no significant difference in age, PSA, or biopsy Gleason scores between patients who underwent adjusted versus nonadjusted surgery [3]. Table 2 summarizes the surgical procedures in both groups.

**Table 2:** Overview of surgical procedures in the non-MRI and MRI groups

	Non-MRI (*n* = 216)		MRI (*n* = 222)		Difference
	*n*	%	*n*	%	% (95% CI)
Bilateral nerve-sparing surgery	73	34	60	27	7 (–2 to 15)
Unilateral nerve-sparing surgery	71	33	80	36	3 (–6 to 12)
Non–nerve-sparing surgery	72	33	82	37	4 (–3 to 23)
Pelvic lymph node dissection	31	14	36	16	2 (–5 to 9)

CI = confidence interval; MRI = magnetic resonance imaging.

The PSM rate was 23% in the non-MRI group and 19% in the MRI group (*p* = 0.4). Among cases with PSMs, the responsible tumor was detected on MRI in 92% of cases. The base and apex were the most common locations for PSMs, with a trend toward a 12% increased risk at the ventral apex in the non-MRI group (95% CI: –3 to 23) [3].

PLND was performed in 15% of the total cohort (67/438 patients), of whom 73% (49/67 patients) were in the high-risk group. There was no difference in the PLND rate between the non-MRI and MRI groups. PLND was positive for lymph node metastases in 12% (eight of 67 patients): three patients in the non-MRI group and five in the MRI group. Table 3 summarizes the pathological characteristics of both groups.

**Table 3:** Postoperative pathological characteristics of 438 patients randomized to the non-MRI or MRI group before radical prostatectomy

	Non-MRI (*n* = 216)		MRI (*n* = 222)	
	*n*	%	*n*	%
Gleason grade group in specimen				
1	59	27	57	26
2	83	38	87	39
3	44	20	48	22
4	23	11	22	10
5	7	3	8	4
Pathological tumor stage				
pT2	111	51	103	46
pT3	105	49	119	54
Pathological lymph node status				
pN+	3	1.4	5	2.3
Positive surgical margin	49	23	43	19
	Median	IQR	Median	IQR
Index tumor volume in specimen (ml)	2.2	1.0–4.5	2.3	1.0–4.3

IQR = interquartile range; MRI = magnetic resonance imaging; pN+ = positive lymph node metastases; pT = pathological tumor stage.

During a median follow-up time of 111 mo (95% CI: 104–118), 75 patients had an event (persistent disease or BCR) in the non-MRI group and 77 in the MRI group. The median time to event was 18 mo (95% CI: 8–29) in the non-MRI group and 18 mo (95% CI: 11–25) in the MRI group. Persistent disease occurred in 26 patients in the non-MRI group and 23 patients in the MRI group. BCR occurred in 49 patients in the non-MRI group and 54 patients in the MRI group.

**Fig. 1:**
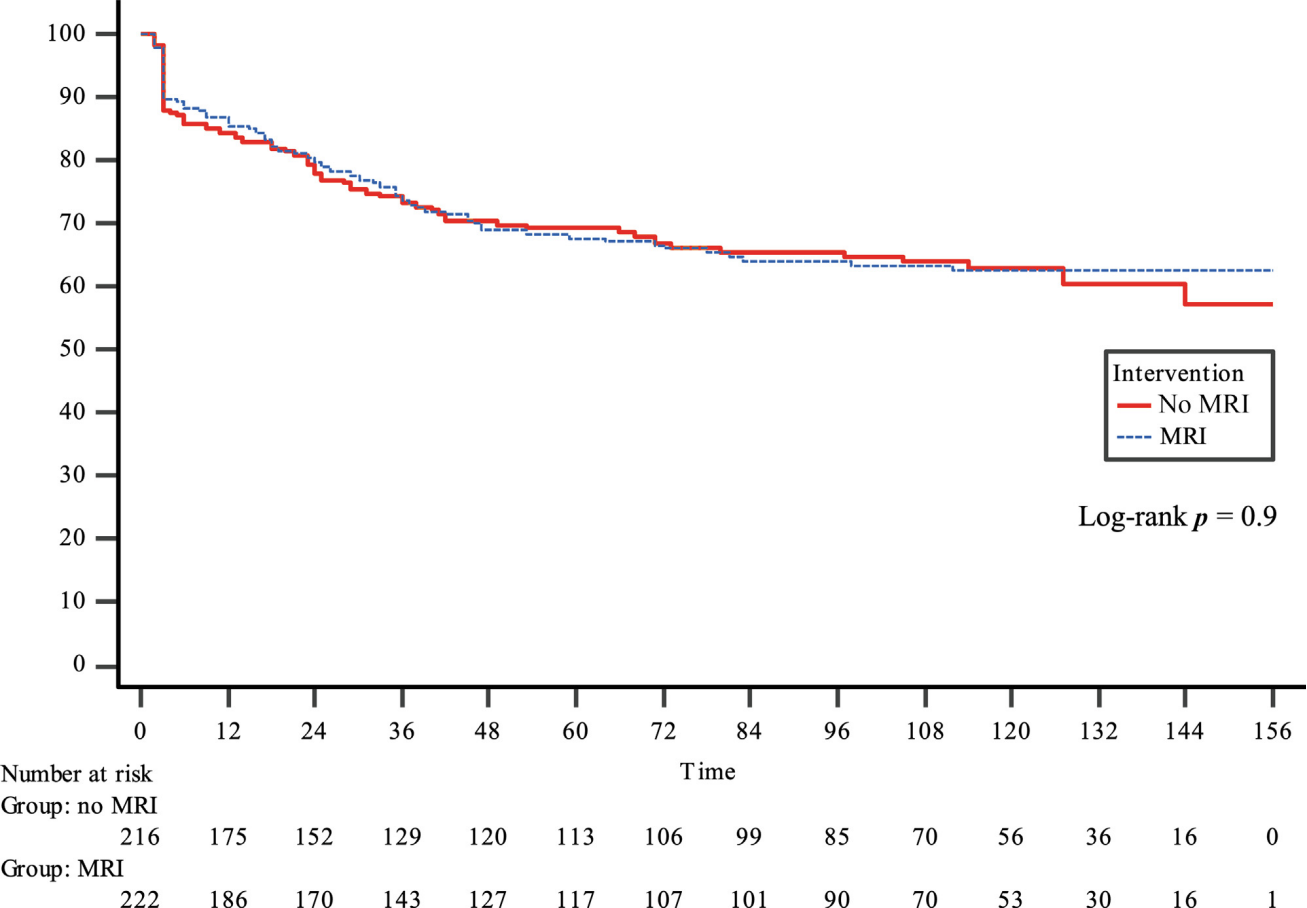
Kaplan-Meier curves for disease-free survival in patients randomized to no MRI or MRI prior to radical prostatectomy. MRI = magnetic resonance imaging.

**Fig. 2:**
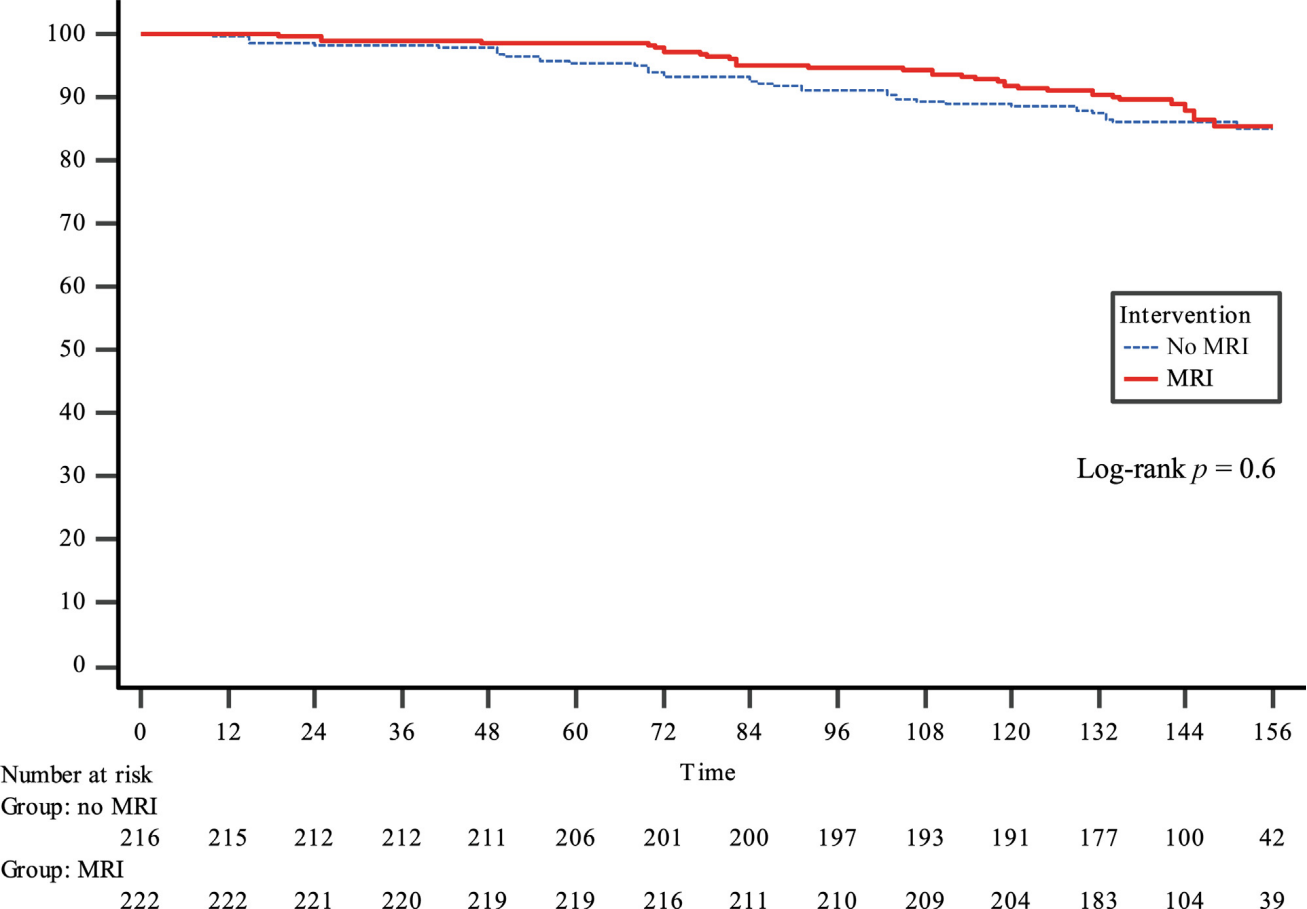
Kaplan-Meier curves for overall survival in patients randomized to no MRI or MRI prior to radical prostatectomy. MRI = magnetic resonance imaging.

The DFS rates at 3, 5, and 12 yr were 73% (95% CI: 69–77), 68% (95% CI: 64–73), and 60% (95% CI: 54–65), respectively (Fig. 1). Importantly, preoperative MRI showed no benefit on DFS (HR 0.97, 95% CI: 0.71–1.34), and this finding was consistent across all clinical subgroups (Table 4). The OS rates at 3, 5, and 12 yr were 99% (95% CI: 97–99), 97% (95% CI: 95–99), and 87% (95% CI: 84–90), respectively. Similarly, preoperative MRI showed no benefit on OS (HR 0.86, 95% CI: 0.52–1.40; Fig. 2).

**Table 4:** Univariate Cox regression subgroup analysis comparing the risk of persistent disease or biochemical recurrence and death by any cause between the MRI group and the non-MRI (reference) group

	Risk of persistent disease or biochemical recurrence			Risk of death by any cause		
	Hazard ratio	95% CI	*p* value a	Hazard ratio	95% CI	*p* value a
All	0.97	0.71–1.34		0.86	0.52–1.40	
Pathological tumor stage			0.9			0.6
pT2	0.87	0.46–1.65		0.72	0.31–1.69	
pT3	0.89	0.62–1.29		0.93	0.49–1.77	
D’Amico risk group			0.7			0.8
Low	0.92	0.39–2.17		0.80	0.29–2.21	
Intermediate	1.03	0.62–1.69		1.15	0.54–2.46	
High	1.09	0.68–1.75		0.59	0.22–1.60	
Gleason grade group in specimen			0.5			0.8
1	0.88	0.30–2.63		0.40	0.08–2.08	
2	1.06	0.60–1.90		0.86	0.38–1.94	
3	0.99	0.57–1.72		1.61	0.59–4.44	
4	0.84	0.40–1.75		1.01	0.25–4.05	
5	0.69	0.24–2.00		0.19	0.02–1.68	
Clinical tumor stage			0.3			0.9
cT1	0.85	0.54–1.35		0.87	0.43–1.73	
cT2	1.09	0.68–1.76		0.95	0.43–2.13	
cT3	1.04	0.30–3.56		0.30	0.03–3.30	

CI = confidence interval; cT = clinical tumor stage; MRI = magnetic resonance imaging; pT = pathological tumor stage.

a *p* value for interaction.

## 4 Discussion

This study is the first randomized controlled trial to assess the effect of preoperative MRI on long-term oncological outcomes after RALP. Over a 12-yr follow-up period, we observed no effect on DFS (HR 0.97, 95% CI: 0.71–1.34) or OS (HR 0.86, 95% CI: 0.52–1.40), with consistent results across all clinical subgroups (Table 4).

To date, only two studies, by Joyce et al. [1] and Patel et al., [2] have evaluated the effect of MRI on long-term oncological outcomes. Our findings are consistent with those of Joyce et al., [1] who investigated the effect of preoperative MRI and found no improvement in BCR in the MRI group (HR 1.21, 95% CI: 0.8–1.7). In contrast, Patel et al., [2] who examined prebiopsy MRI, reported a lower risk of BCR in the MRI group (HR 0.64, 95% CI: 0.64–0.99). The discrepancy in the findings may be attributed to the timing of the MRI (prebiopsy vs preoperative) or incidental variation, as both studies are retrospective and nonrandomized.

In our study population, 26% of the patients had low-risk disease and many of these patients would undergo active surveillance today. However, 50% and 25% of the patients had intermediate- and high-risk disease, respectively. This is similar to or higher than the related findings of other studies and shows that the majority of our study population remains relevant to the current practice [1,7].

Our institution has consistently used a biparametric MRI protocol for tumor detection and staging, whereas current recommendations favor slightly higher in-plane resolution (0.6 mm vs 0.9 mm in our study) and dynamic contrast enhancement as part of a standardized multiparametric MRI (mpMRI) protocol. Although mpMRI remains the standard, there has been a shift toward biparametric MRI for detection, with several studies showing equivalent cancer detection rates and staging results [[8], [9], [10], [11], [12]]. In our original study, preoperative MRI detected 92% of tumors responsible for PSMs, with sensitivity of 72% and specificity of 65% for detecting T3 disease [3]. Despite technological advances, recent studies using mpMRI with higher in-plane resolution report sensitivity and specificity for detecting EPE at approximately 53–61% and 86–87%, respectively, showing that MRI staging results remain largely unchanged [13,14].

Although Prostate Imaging Reporting and Data System (PI-RADS) has become a standard tool for assessing the risk of significant prostate cancer prior to biopsy, it was introduced after the completion of our study. Since all patients in our cohort had biopsy-confirmed prostate cancer, PI-RADS would not have been relevant to their workup.

MRI alone does not improve surgical or oncological outcomes unless its findings are actively integrated into surgical planning. In our original study, MRI influenced surgical decisions in 27% of cases, consistently resulting in fewer nerve-sparing surgeries [3]. These adjustments were primarily driven by radiological evidence of more advanced disease, as 87% of patients who underwent altered surgery were classified as being at radiological stage T3. Although the MRI group underwent fewer nerve-sparing (non-MRI group: 34% and MRI group: 27%) and more non–nerve-sparing (non-MRI group: 33% and MRI group: 37%) surgical procedures, the observed effect was less than the 27% reported by the surgeons, and the difference was not statistically significant (Table 3). This discrepancy highlights a potential source of a bias in studies lacking a control arm without MRI.

In comparison, Joyce et al [1] reported similar rates of bilateral nerve-sparing surgery in both groups: 75% in the non-MRI group and 76% in the MRI group. In their study, 98% exhibited cT1 disease, and the median tumor volume was about 1.3 ml for both groups. In our study, the rate of cT1 disease was considerably lower (∼50% in both groups), and the median tumor volume was notably larger (2.2–2.3 ml) [3]. These differences may account for the lower rates of nerve-sparing surgery observed in our cohort. In contrast, Patel et al [2] found that patients who underwent MRI were more likely to receive nerve-sparing surgery (non-MRI group: 60% vs MRI group: 70%). This difference may be explained by a higher prevalence of cT1 disease in the MRI group (90%) than in the non-MRI group (79%). A meta-analysis reported that MRI altered the surgical procedure in 37% of cases, leading to fewer nerve-sparing surgeries in 21% and more nerve-sparing surgeries in 16% [15]. However, most of the included studies lacked a comparative cohort without MRI, raising concern about a bias.

In the studies by Joyce et al [1] and Patel et al [2], most patients in both groups underwent PLND. Although the rate of PLND was quite low in our study (15%), it was similar in both groups and therefore unlikely to have masked any differences in outcomes. Further, the therapeutic benefit of PLND is uncertain, and the impact of these variations on long-term oncological outcomes remains unclear [16].

A PSM is one of the strongest independent predictors of BCR after radical prostatectomy. Although our study indicated that MRI contributed to wider resections, there was no significant improvement in PSMs (non-MRI group: 23% vs MRI group: 19%, *p* = 0.4) [3]. In comparison, five studies, including those by Joyce et al [1] and Patel et al [2], failed to show a significant reduction in PSM rates for the MRI group [3,17,18]. Conversely, two studies reported a significant reduction in PSM rates of 7% and 12% [19,20]. A meta-analysis, including most of the abovementioned studies, estimated a 5% reduction in the PSM rate (*p* < 0.001) in patients who underwent preoperative MRI, suggesting that the actual effect of MRI could be around this figure [2]. However, the retrospective and nonrandomized nature of these studies raises concerns about a potential bias. Although, we originally found a significant reduction of PSMs in the MRI group for patients with cT1, our follow-up study did not demonstrate an improvement in DFS or OS [3]. This highlights the need for caution when interpreting results from subgroup analyses lacking follow-up data, as noted in the original study. Recent studies using 3D models and augmented reality systems have demonstrated reduced PSM rates [21,22]. Although promising, more studies, including randomized controlled trials, are needed to evaluate both immediate and long-term effects of such complex interventions.

In our study, the apex and base were high-risk sites for PSMs, and the non-MRI group showed a trend toward a higher risk (13%) at the ventral apex compared with the MRI group. Although the clinical impact of PSM location remains controversial, a meta-analysis reported that PSMs at the anterior margin were associated with a higher risk of recurrence [23].

In our study, 11% exhibited persistent disease, as shown by the initial steep decline in the DFS curves in Fig. 1, resulting in a 5-yr DFS rate of 68%. In contrast, the studies by Joyce et al [1] and Patel et al [2] did not account for persistent disease and reported a 5-yr BCR-free survival rate of approximately 80%. This aligns with our results when cases of persistent disease are excluded. Patients with persistent disease and BCR should be included when evaluating oncological outcomes, as both conditions indicate treatment failure.

Our study had a median follow-up of 111 mo and offers a more comprehensive perspective on long-term outcomes compared with the 25 mo of median follow-up in the study by Patel et al [2]. Interestingly, Joyce et al [1] reported a median follow-up of 62 mo (5.2 yr) in the non-MRI group and 19 mo (1.6 yr) for the MRI group, raising concerns about the comparability of the two groups. A longer follow-up period in the MRI group might have revealed a higher recurrence rate.

### 4.1 Limitations

It is important to note that this study was designed to assess the effect of MRI on surgical margin, and not on long-term oncological outcomes. As with all secondary outcomes, it is necessary to interpret these results with caution, especially given the wide 95% CIs of the HRs. Since the time we started conducting the trial, the clinical pathway for prostate cancer has undergone significant changes with the introduction of prebiopsy MRI. Several studies have shown that prebiopsy MRI and targeted biopsies improve the detection of clinically significant cancers and reduce overdiagnosis compared with systematic biopsies without MRI [4,[24], [25], [26]]. However, the long-term impact of prebiopsy MRI remains uncertain, and our findings reinforce the need to investigate the effect of the current MRI pathway on long-term surgical and oncological outcomes. Furthermore, the potential impact of image quality, postbiopsy artifacts, and our use of 3D T2-weighted images with slightly lower in-plane resolution (0.9 mm vs the recommended 0.6 mm) may have influenced staging results. However, the observed staging accuracy aligns with the findings in the existing literature [27].

## 5 Conclusions

During a 12-yr follow-up of a randomized controlled trial in patients who underwent RALP, we observed no statistically significant difference in DFS or OS between patients who received preoperative MRI and those who did not. Nonetheless, the wide CIs reflect considerable uncertainty. As prebiopsy MRI has become the standard practice, our findings highlight the need to assess its impact on long-term surgical and oncological outcomes.

  ***Author contributions*:** Daniyal Noor had full access to all the data in the study and takes responsibility for the integrity of the data and the accuracy of the data analysis.

  *Study concept and design*: Rud, Noor, Baco, Eri.

*Acquisition of data*: Rud, Noor, Berge, Eri, Baco.

*Analysis and interpretation of data*: Rud, Noor, Lauritzen.

*Drafting of the manuscript*: Noor, Rud, Lauritzen, Galtung.

*Critical revision of the manuscript for important intellectual content*: Rud, Baco, Lauritzen, Berge, Galtung, Jacewicz, Eri.

*Statistical analysis*: Rud, Noor, Lauritzen.

*Obtaining funding*: None.

*Administrative, technical, or material support*: None.

*Supervision*: Rud, Baco.

*Other*: None.

  ***Financial disclosures:*** Daniyal Noor certifies that all conflicts of interest, including specific financial interests and relationships and affiliations relevant to the subject matter or materials discussed in the manuscript (eg, employment/affiliation, grants or funding, consultancies, honoraria, stock ownership or options, expert testimony, royalties, or patents filed, received, or pending), are the following: None.

  ***Funding/Support and role of the sponsor*:** None.

## References

[1] Joyce D.D., Soligo M., Morlacco A. (2023). Effect of preoperative multiparametric magnetic resonance imaging on oncologic and functional outcomes following radical prostatectomy. Eur Urol Open Sci.

[2] Patel H.D., Okabe Y., Rac G. (2023). MRI versus non-MRI diagnostic pathways before radical prostatectomy: impact on nerve-sparing, positive surgical margins, and biochemical recurrence. Urol Oncol.

[3] Rud E., Baco E., Klotz D. (2015). Does preoperative magnetic resonance imaging reduce the rate of positive surgical margins at radical prostatectomy in a randomised clinical trial?. Eur Urol.

[4] Rouvière O., Puech P., Renard-Penna R. (2019). Use of prostate systematic and targeted biopsy on the basis of multiparametric MRI in biopsy-naive patients (MRI-FIRST): a prospective, multicentre, paired diagnostic study. Lancet Oncol.

[5] Rud E., Klotz D., Rennesund K. (2014). Detection of the index tumour and tumour volume in prostate cancer using T2‐weighted and diffusion‐weighted magnetic resonance imaging (MRI) alone. BJU Int.

[6] Rud E., Klotz D., Rennesund K. (2015). Preoperative magnetic resonance imaging for detecting uni- and bilateral extraprostatic disease in patients with prostate cancer. World J Urol.

[7] Falagario U.G., Abbadi A., Remmers S. (2023). Biochemical recurrence and risk of mortality following radiotherapy or radical prostatectomy. JAMA Netw Open.

[8] UroToday. EAU 2024: comparison of biparametric and multiparametric MRI for prostate cancer detection: the PRIME study. https://www.urotoday.com/conference-highlights/eau-2024/eau-2024-prostate-cancer/150992-eau-2024-comparison-of-biparametric-and-multiparametric-mri-for-prostate-cancer-detection-the-prime-study.html.

[9] Asif A., Nathan A., Ng A. (2023). Comparing biparametric to multiparametric MRI in the diagnosis of clinically significant prostate cancer in biopsy-naive men (PRIME): a prospective, international, multicentre, non-inferiority within-patient, diagnostic yield trial protocol. BMJ Open.

[10] Zawaideh J.P., Sala E., Shaida N. (2020). Diagnostic accuracy of biparametric versus multiparametric prostate MRI: assessment of contrast benefit in clinical practice. Eur Radiol.

[11] Xu L., Zhang G., Shi B. (2019). Comparison of biparametric and multiparametric MRI in the diagnosis of prostate cancer. Cancer Imaging.

[12] Caglic I., Sushentsev N., Shah N., Warren A.Y., Lamb B.W., Barrett T. (2021). Comparison of biparametric versus multiparametric prostate MRI for the detection of extracapsular extension and seminal vesicle invasion in biopsy naïve patients. Eur J Radiol.

[13] Zhang F., Liu C.-L., Chen Q., Shao S.-C., Chen S.-Q. (2019). Accuracy of multiparametric magnetic resonance imaging for detecting extracapsular extension in prostate cancer: a systematic review and meta-analysis. Br J Radiol.

[14] Thaiss W.M., Moser S., Hepp T. (2022). Head-to-head comparison of biparametric versus multiparametric MRI of the prostate before robot-assisted transperineal fusion prostate biopsy. World J Urol.

[15] Kozikowski M., Malewski W., Michalak W., Dobruch J. (2019). Clinical utility of MRI in the decision-making process before radical prostatectomy: systematic review and meta-analysis. PLoS One.

[16] Fujimoto N., Shiota M., Tomisaki I., Minato A., Yahara K. (2019). Reconsideration on clinical benefit of pelvic lymph node dissection during radical prostatectomy for clinically localized prostate cancer. Urol Int.

[17] Gietelink L., Jansen B.H.E., Oprea-Lager D.E., Nieuwenhuijzen J.A., Vis A.N. (2022). Preoperative multiparametric MRI does not lower positive surgical margin rate in a large series of patients undergoing robot-assisted radical prostatectomy. J Robot Surg.

[18] Druskin S.C., Liu J.-J., Young A. (2017). Prostate MRI prior to radical prostatectomy: effects on nerve sparing and pathological margin status. Res Rep Urol.

[19] Jäderling F., Akre O., Aly M. (2019). Preoperative staging using magnetic resonance imaging and risk of positive surgical margins after prostate-cancer surgery. Prostate Cancer Prostatic Dis.

[20] Schiavina R., Bianchi L., Borghesi M. (2018). MRI displays the prostatic cancer anatomy and improves the bundles management before robot-assisted radical prostatectomy. J Endourol.

[21] Porpiglia F., Checcucci E., Amparore D. (2019). Three-dimensional elastic augmented-reality robot-assisted radical prostatectomy using hyperaccuracy three-dimensional reconstruction technology: a step further in the identification of capsular involvement. Eur Urol.

[22] Checcucci E., Pecoraro A., Amparore D. (2022). The impact of 3D models on positive surgical margins after robot-assisted radical prostatectomy. World J Urol.

[23] John A., Milton T., Gupta A. (2025). Impact of positive surgical margin location after radical prostatectomy: a network meta-analysis. World J Urol.

[24] Zhang J., Zhu A., Sun D., Guo S., Zhang H., Liu S. (2020). Is targeted magnetic resonance imaging/transrectal ultrasound fusion prostate biopsy enough for the detection of prostate cancer in patients with PI-RADS ≥3: results of a prospective, randomized clinical trial. J Cancer Res Ther.

[25] Labra A., González F., Silva C., Franz G., Pinochet R., Gupta R.T. (2020). MRI/TRUS fusion vs. systematic biopsy: intra-patient comparison of diagnostic accuracy for prostate cancer using PI-RADS v2. Abdom Radiol.

[26] Elwenspoek M.M.C., Sheppard A.L., McInnes M.D.F. (2019). Comparison of multiparametric magnetic resonance imaging and targeted biopsy with systematic biopsy alone for the diagnosis of prostate cancer. JAMA Netw Open.

[27] de Rooij M., Hamoen E.H.J., Fütterer J.J., Barentsz J.O., Rovers M.M. (2014). Accuracy of multiparametric MRI for prostate cancer detection: a meta-analysis. Am J Roentgenol.

